# Supplementary material for: 3D visualization of additive occlusion and tunable full-spectrum fluorescence in calcite
Source: Nat Commun. 2016 Nov 18;7:13524. doi: 10.1038/ncomms13524 (PMC5120221; doi:10.1038/ncomms13524)
Supplement: Supplementary Information — Supplementary Figures 1-19 and Supplementary Tables 1-2. [file ncomms13524-s1.pdf]

Supplementary Figures

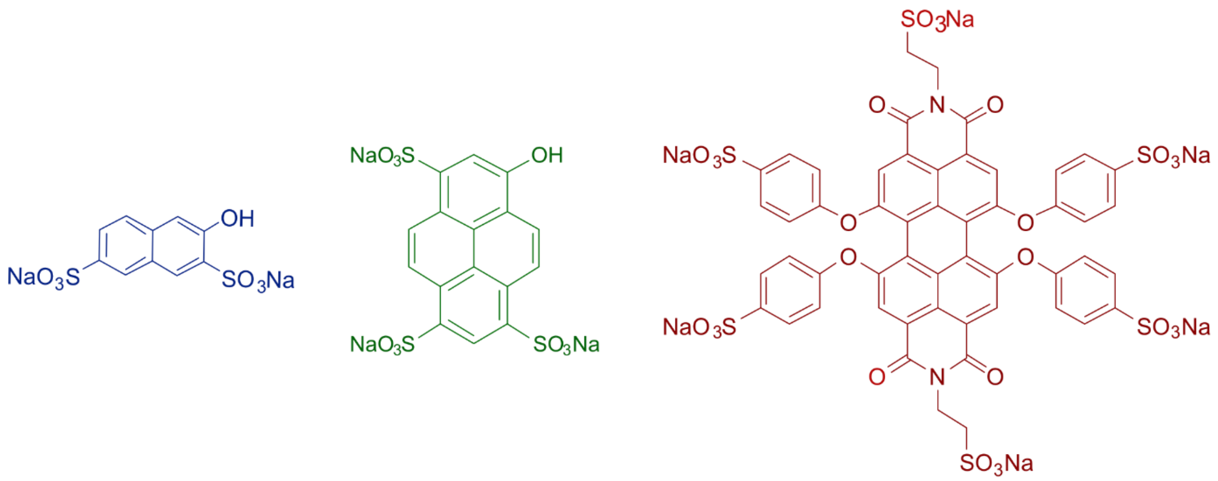

Supplementary Figure 1: Molecular structures of fluorescent dyes used for generating host-guest calcite-based fluorophores: HNDS (BLUE), HPTS (GREEN) and RED.

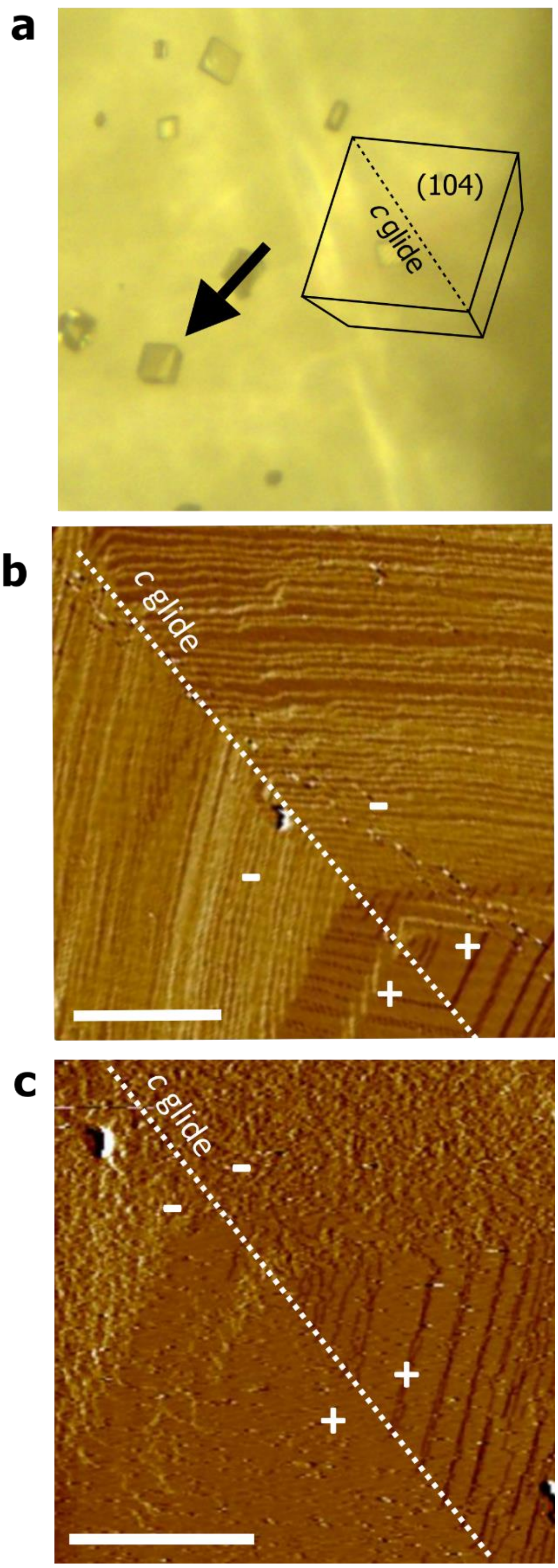

Supplementary Figure 2: **a**) Optical micrograph of calcite seed and schematic diagram depicting orientation of the crystal and situation of the c glide. **b**) AFM image obtained in contact mode of calcite seed surface after overgrowth without GREEN; and **c**) AFM image obtained in contact mode of same growth hillock after addition of GREEN to growth solution showing preferential (although not exclusive) interaction with acute steps. Scale bars: 1 µm

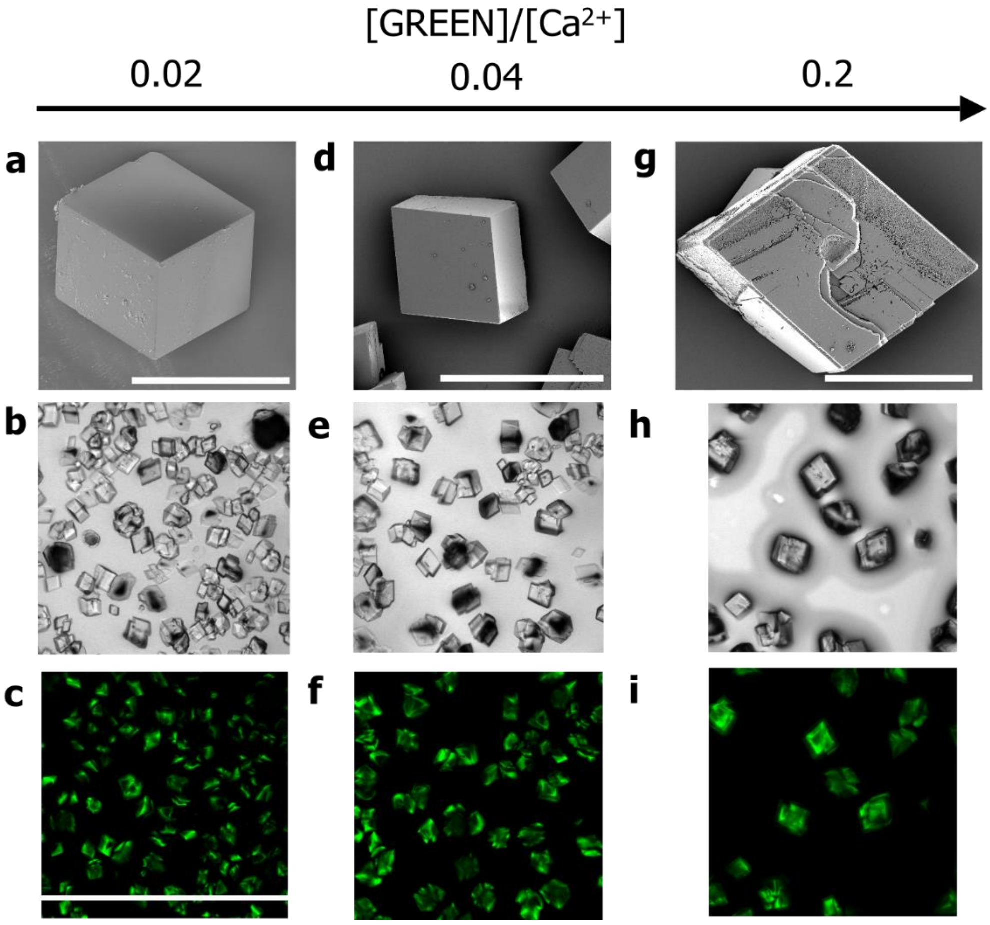

Supplementary Figure 3: GREEN/calcite composites from $[Ca^{2+}] = [CO_3^{2-}] = 5$ mM conditions, grown in the presence of different $[GREEN]/[Ca^{2+}]$ (**a-c** = 0.02, **d-f** = 0.04 and **g-i** = 0.2) as detailed by SEM (**a, d, g**), optical (**b, e, h**) and confocal fluorescence microscopy (**c, f, i**). Scale bars: a) 40 µm, d) 50 µm, g) 30 µm, c) 450 µm applies for all confocal and optical images.

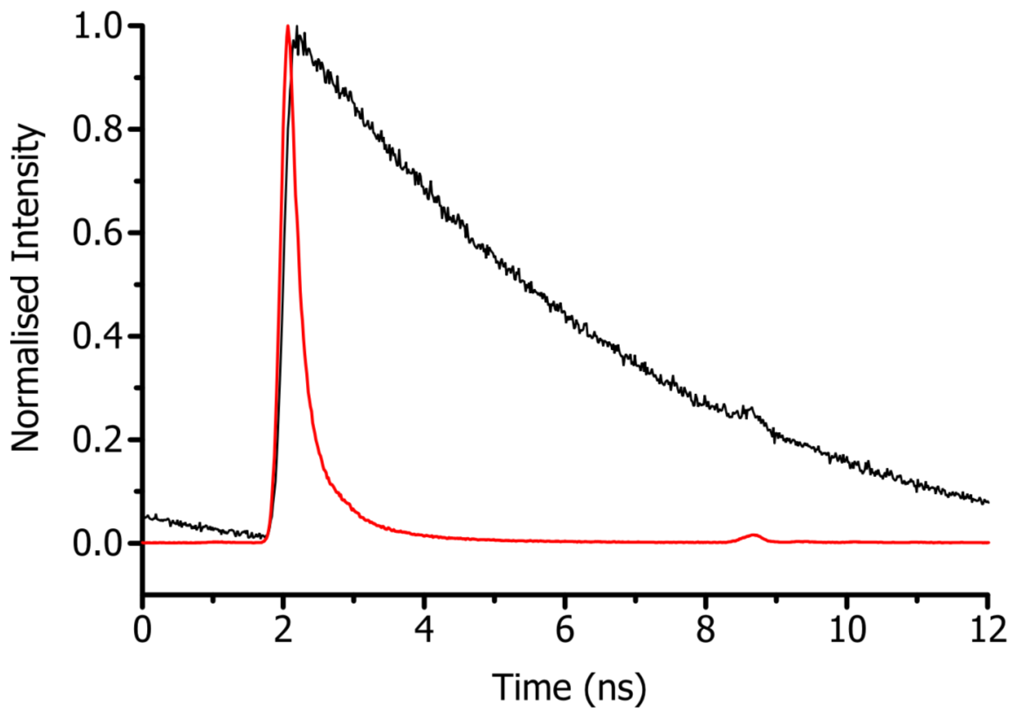

Supplementary Figure 4: FLIM decay curves for dry (red) and aqueous solution (black) of GREEN. Calculated lifetimes τ = 0.6 and 5.4 ns respectively.

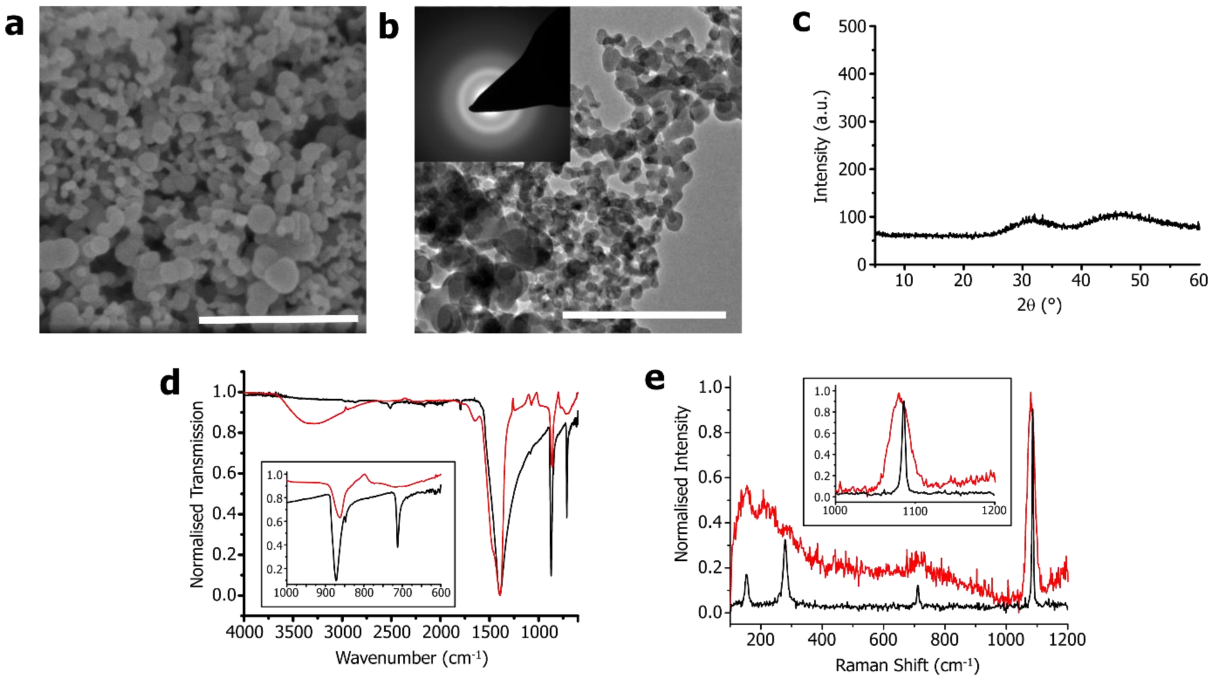

Supplementary Figure 5: **a**) Representative SEM micrograph of ACC formed by the method stated in this report. **b**) Representative TEM micrograph of ACC, with SAED demonstrating absence of diffraction pattern characteristic to crystalline $CaCO_3$ (inset). **c**) pXRD of ACC showing 2 characteristic broad peaks within the range 2θ = 5 – 60°. **d**) FTIR spectra of ACC (black) and calcite (red) for comparison. Characteristic absence of peak at 712 cm^{-1} was used as confirmation of amorphous phase (inset). **e**) Raman spectra of ACC (red) and calcite (black) for comparison. Characteristic broad peak at 1085 cm^{-1} was used as confirmation of amorphous phase (inset). Scale bars: a) 500 µm, b) 350 nm

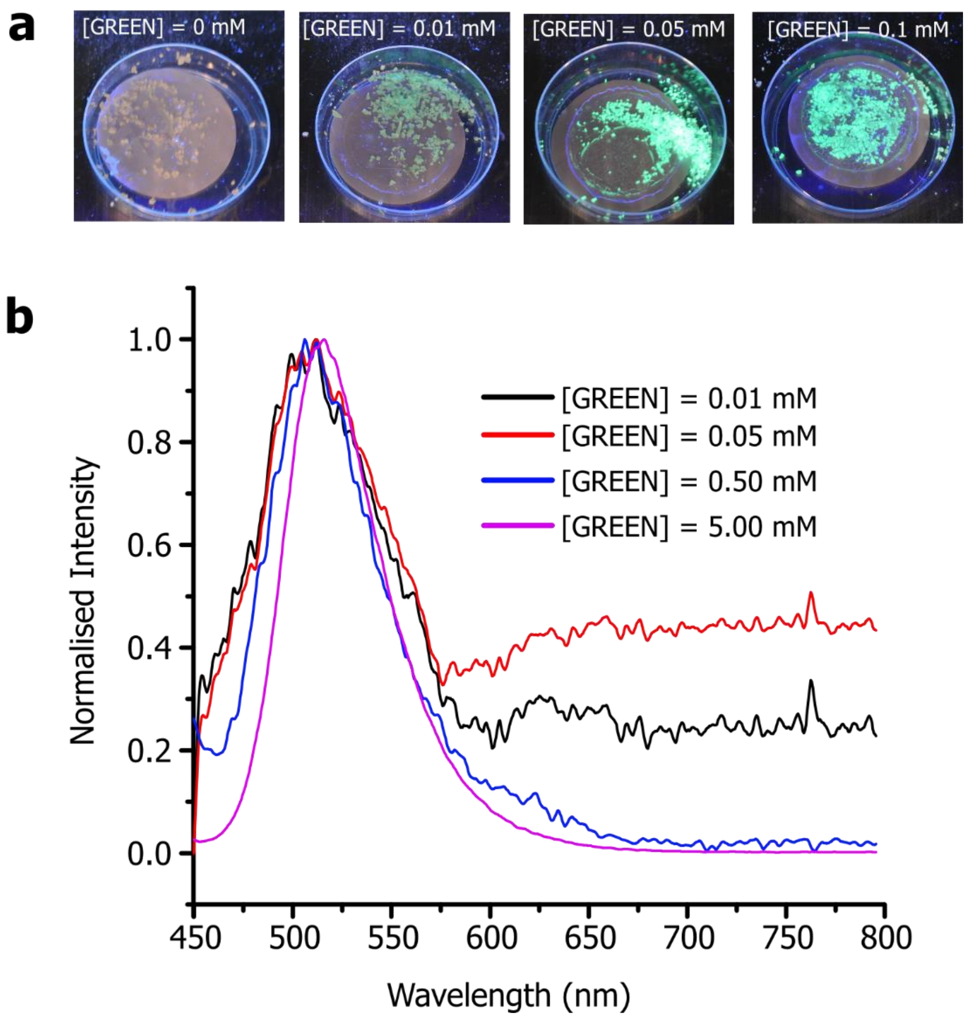

Supplementary Figure 6: Photographs of bulk GREEN/ACC composites of samples excited by UV light (365 nm) with different initial [GREEN] (**a**). Background-removed emission spectra of composites made in the presence different initial [GREEN] indicate no significant change in emissive properties, comparable to that of aqueous dye solutions, particularly regarding secondary peaks at lower energy (**b**).

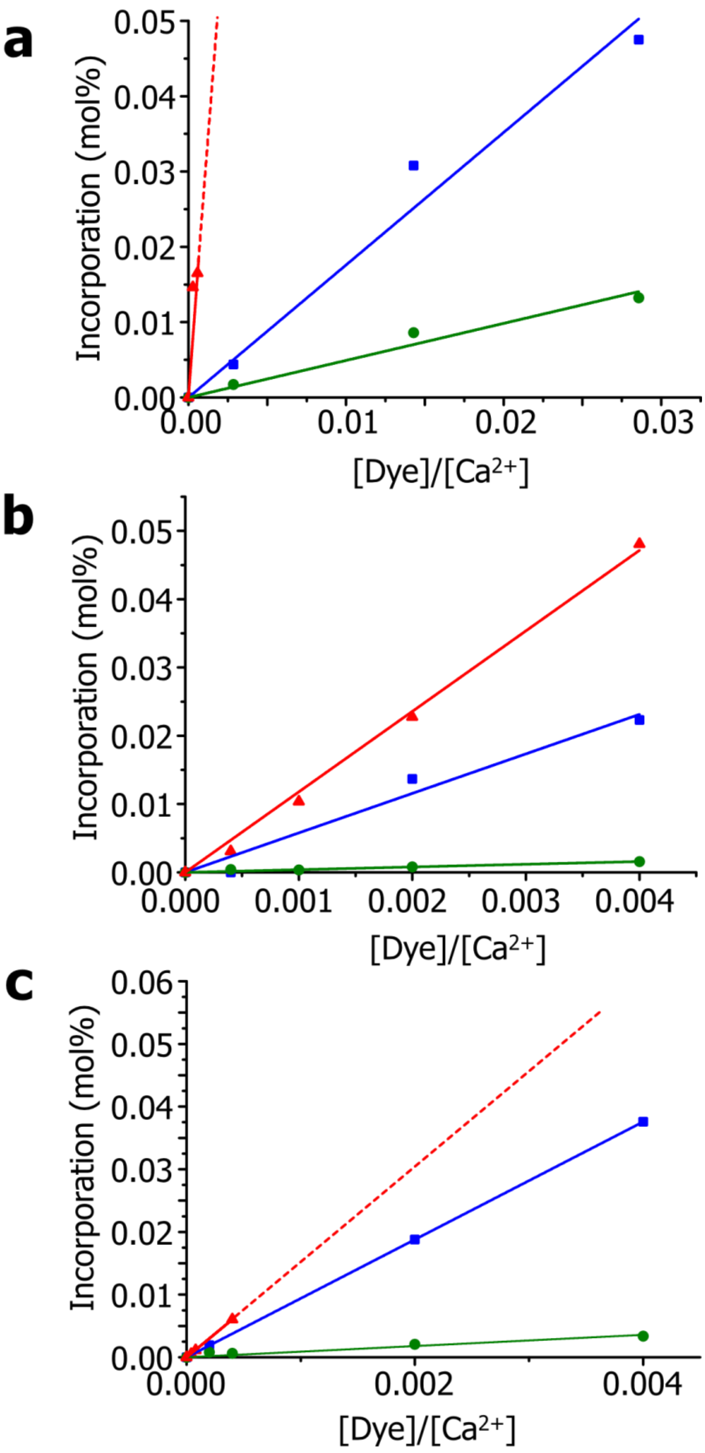

Supplementary Figure 7: Incorporation in mol% vs. initial [Dye]/[Ca^{2+}] plots for BLUE (blue square), GREEN (green circle) and RED (red triangle) under different conditions: **a**) [Ca^{2+}] = [HCO_3^-] = 3.5 mM, **b**) [Ca^{2+}] = [CO_3^{2-}] = 5 mM and **c**) [Ca^{2+}] = [CO_3^{2-}] = 25 mM. Dotted lines represent extrapolated straight line trends.

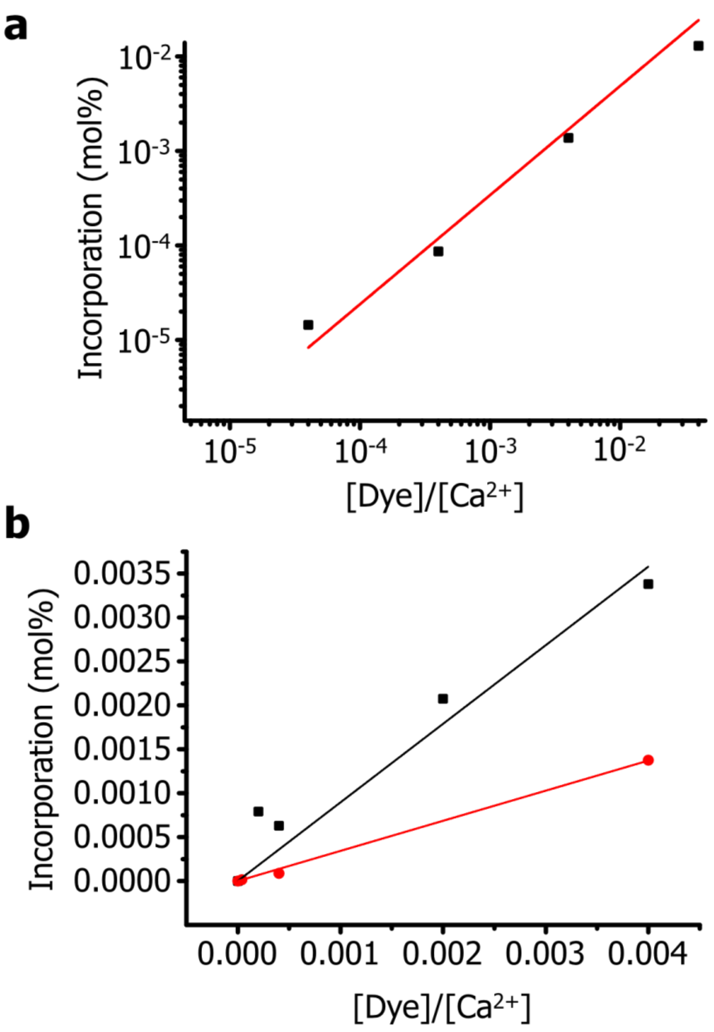

Supplementary Figure 8: **a**) Quantitative analysis of GREEN associated with ACC revealed a linear relationship between mol% incorporation and initial [Dye]/[Ca^{2+}] ratio. **b**) Comparison between calcite samples (Black) and ACC (Red) grown from identical initial conditions revealed the higher propensity for GREEN to incorporate into the crystalline phase.

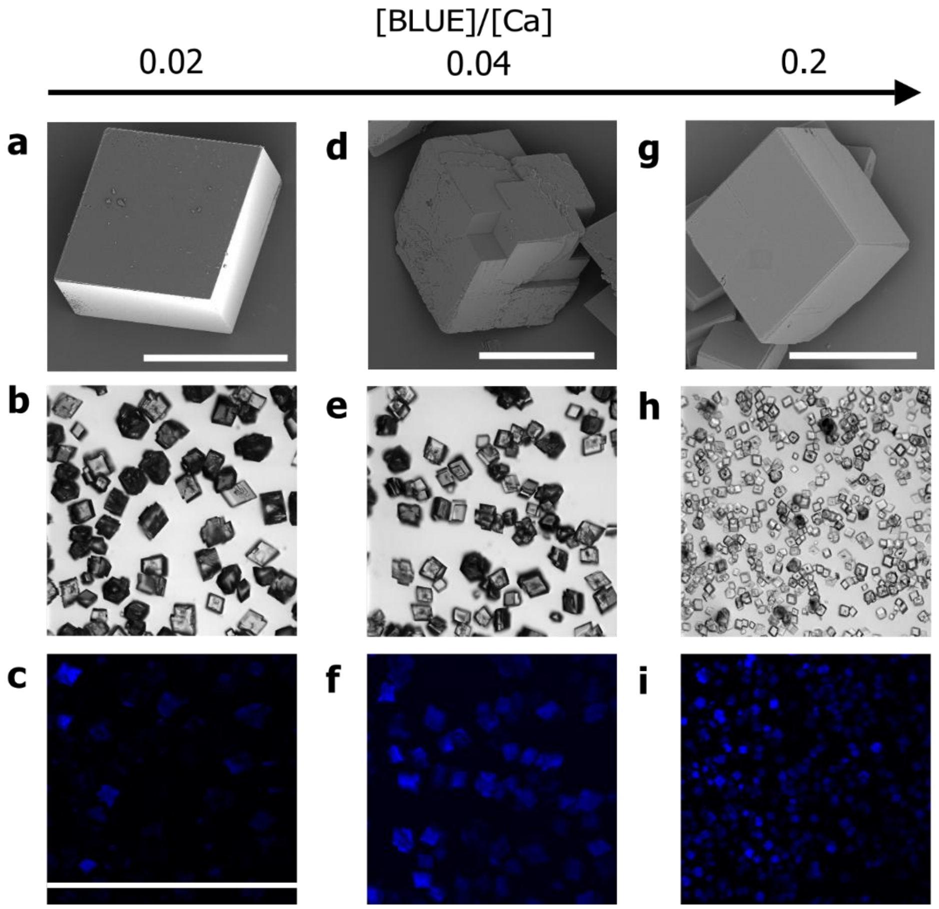

Supplementary Figure 9: BLUE/calcite composites from [Ca^{2+}] = [CO_3^{2-}] = 5 mM conditions, grown in the presence of different [BLUE]/[Ca^{2+}] (**a-c** = 0.02, **d-f** = 0.04 and **g-i** = 0.2) as detailed by SEM (**a, d, g**), optical (**b, e, h**) and confocal fluorescence microscopy (**c, f, i**). Scale bars: a and d) 30 µm, g) 20 µm, c) 450 µm applies to all confocal and optical images.

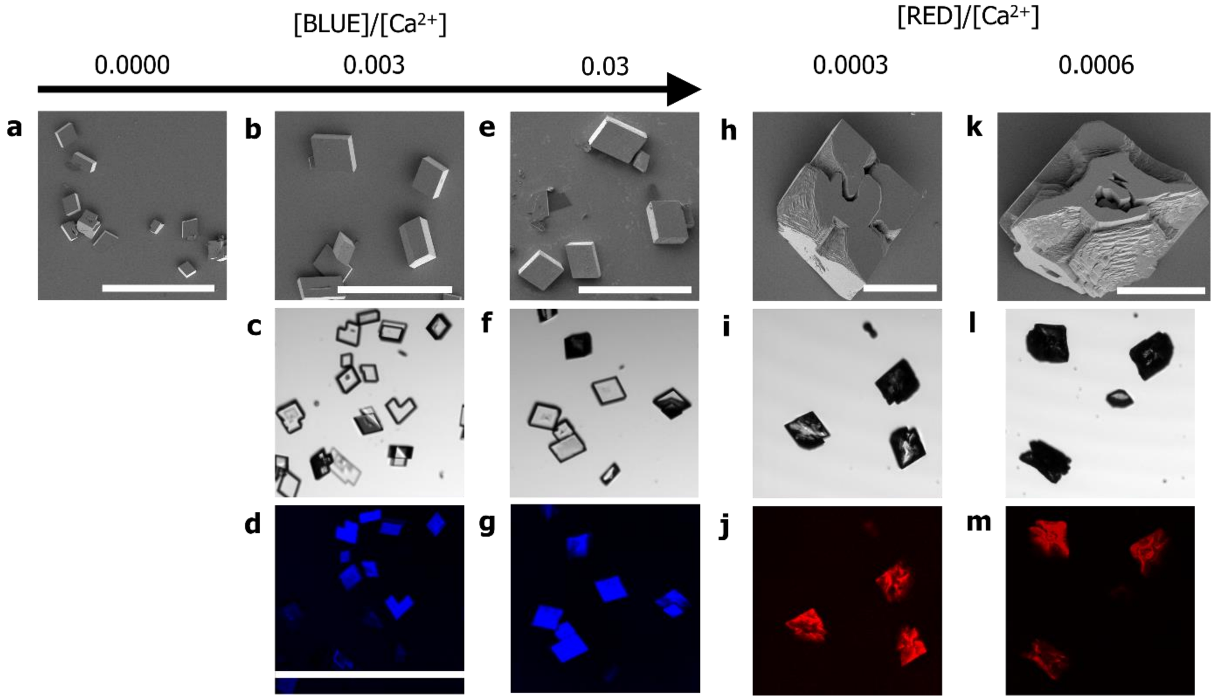

Supplementary Figure 10: BLUE/calcite and RED/calcite composites from [Ca^{2+}] = [HCO_3^-] = 3.5 mM conditions, grown in the presence of different [Dye]/[Ca^{2+}] (**a** = 0, **b-d** = 0.003 BLUE, **e-g** = 0.03 BLUE, **h-j** = 0.0003 RED and **k-m** = 0.0006 RED) as detailed by SEM (**a, b, e, h, k**), optical (**c, f, i, l**) and confocal fluorescence microscopy (**d, g, j, m**). Optical and confocal fluorescence microscopy not shown for [Dye]/[Ca^{2+}] = 0 due to lack of fluorescence (no dye). Scale bars: a) 500 µm, b and e) 200 µm, h and k) 50 µm, d) 450 µm applies to all optical and confocal images.

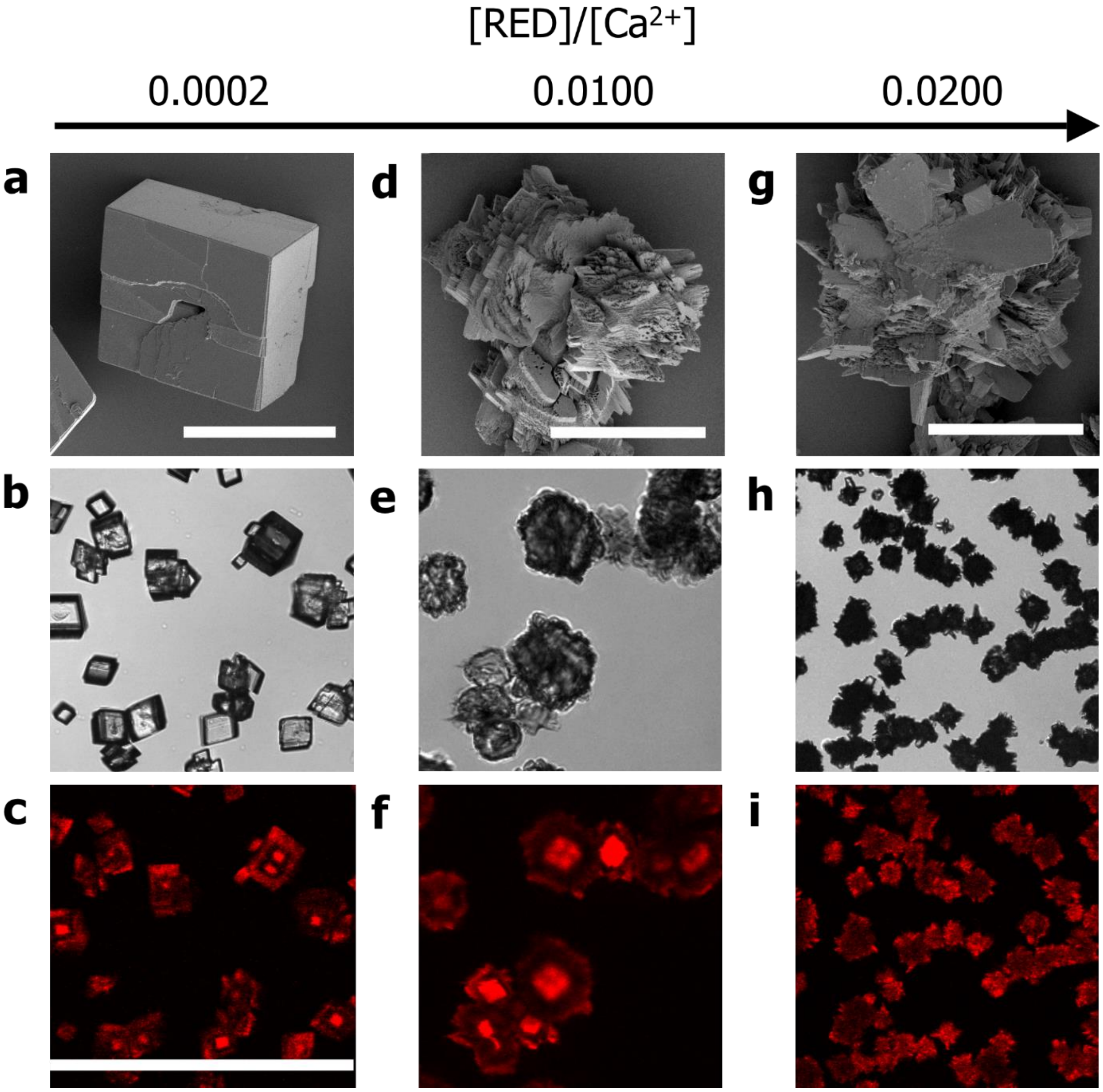

Supplementary Figure 11: RED/calcite composites from $[Ca^{2+}] = [CO_3^{2-}] = 5$ mM conditions, grown in the presence of different [RED]/[Ca] (**a-c** = 0.0002, **d-f** = 0.0400 and **g-i** = 0.0200) as detailed by SEM (**a, d, g**), optical (**b, e, h**) and confocal fluorescence microscopy (**c, f, i**). Scale bars: a) 50 µm, d) 25 µm, g) 20 µm, c) 200 µm applies to all confocal and optical images.

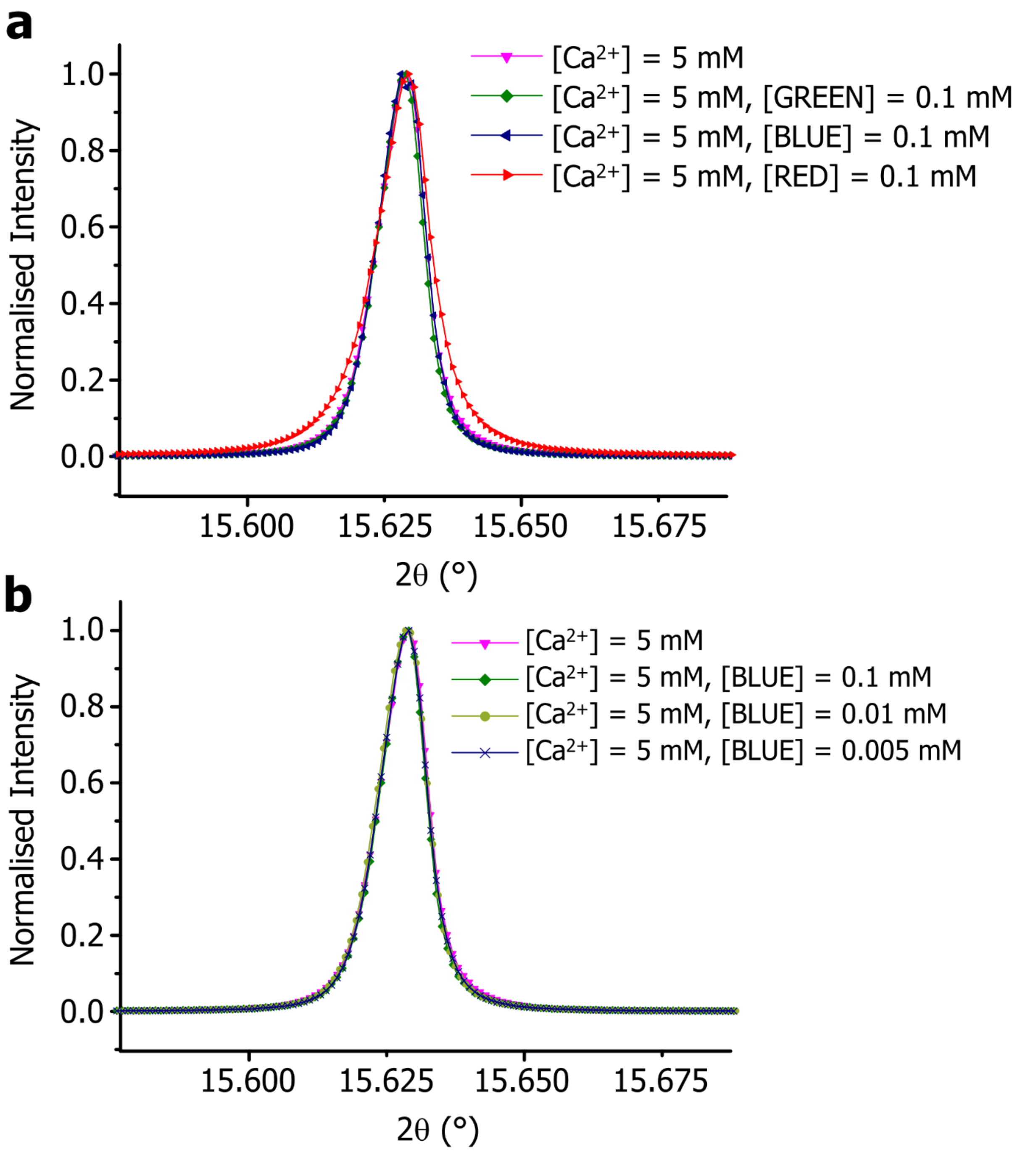

Supplementary Figure 12: High resolution pXRD patterns centered on the reflection attributed to [104] of calcite. **a**) Patterns taken from samples of $[Ca^{2+}]$ = $[CO_3^{2-}\}$ = 5mM with the highest initial [Dye] analysed ([Dye] = 0.1 mM), and therefore highest incorporated dye, showing no significant change to peak centre or peak broadening. **b**) Patterns taken from samples of $[Ca^{2+}]$ = $[CO_3^{2-}\}$ = 5mM with initial [BLUE] = 0, 0.1, 0.01 and 0.005 mM showing no significant change to peak centre or peak broadening in increasing incorporation.

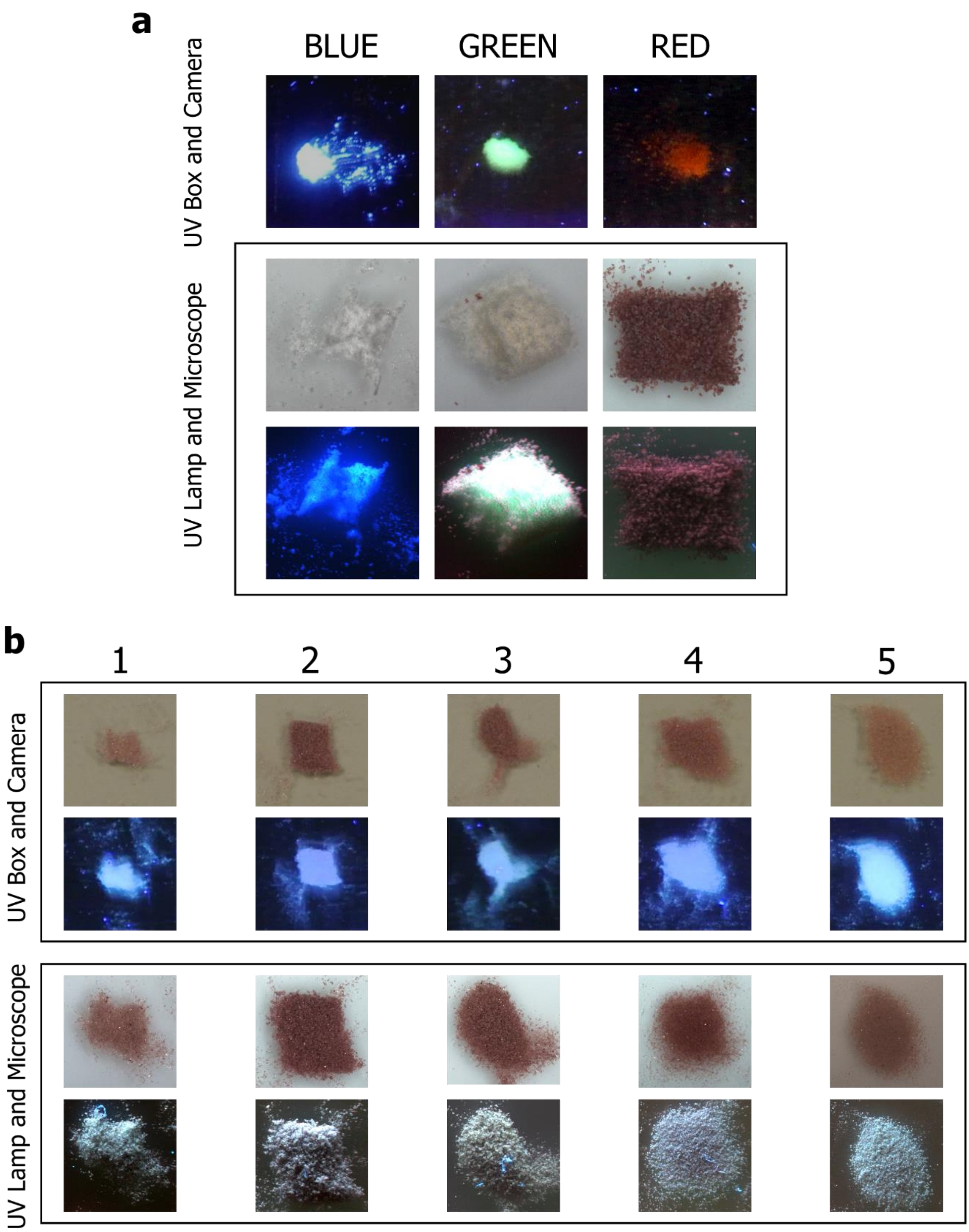

Supplementary Figure 13: Demonstration of bulk fluorescence from dye/composites containing single dyes (**a**) and mixed dyes for full-spectrum white fluorescence (**b**). All fluorescence images were obtained from sample excited under UV light (365 nm). Images were taken from samples excited in UV box with a camera; or excited with a UV lamp on a widefield optical microscope. “White” calcite labels 1-5 correspond to fluorescent dye mixture stipulated in **Supplementary Table 2**.

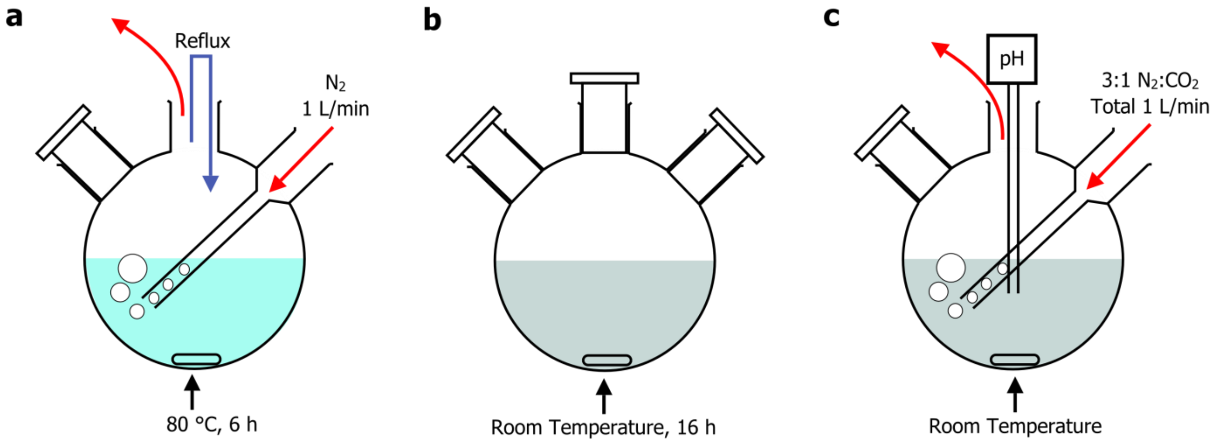

Supplementary Figure 14: Schematic detailing carbonation method for one-pot nanoparticle synthesis. **a**) 50 mL DI water is decarbonated through heating to 80 °C and bubbling with N_2 at 1 L/min under stirring for 6 h. **b**) 0.44 g CaO is added to decarbonated water, the 3-necked flask is stopped at each neck and the slurry is allowed to age under stirring for 16 h at room temperature. **c**) Carbonation with a 3:1 N_2/CO_2 mixture at final flow rate of 1 L/min under stirring at room temperature in an open system. Reaction profile monitored with pH, and terminated by removing gas flow and stopping the flask at pH 7.

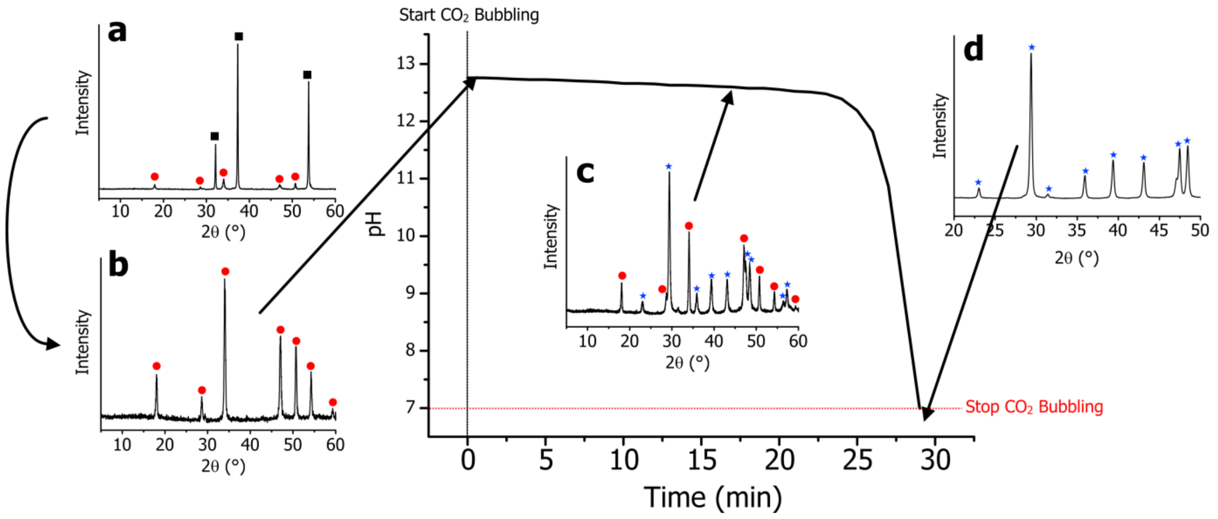

Supplementary Figure 15: Reaction profile of calcite nanoparticles as monitored by pXRD and pH. Initially, CaO (black square, **a**) is hydrolysed to $Ca(OH)_2$ (red circle, **b**). Reaction from $Ca(OH)_2$ was initiated by bubbling of N_2:CO_2 3:1 mixture at 1 L/min at t = 0 (black line). Due to the buffering by OH^-, lowering of pH through CO_2 dissolution was very slow, as $CaCO_3$ formed (**c**) until all $Ca(OH)_2$ had reacted and pH dropped rapidly. Reaction was terminated when pH reached 7 and $CaCO_3$ was the only remaining crystalline phase (red line, **d**).

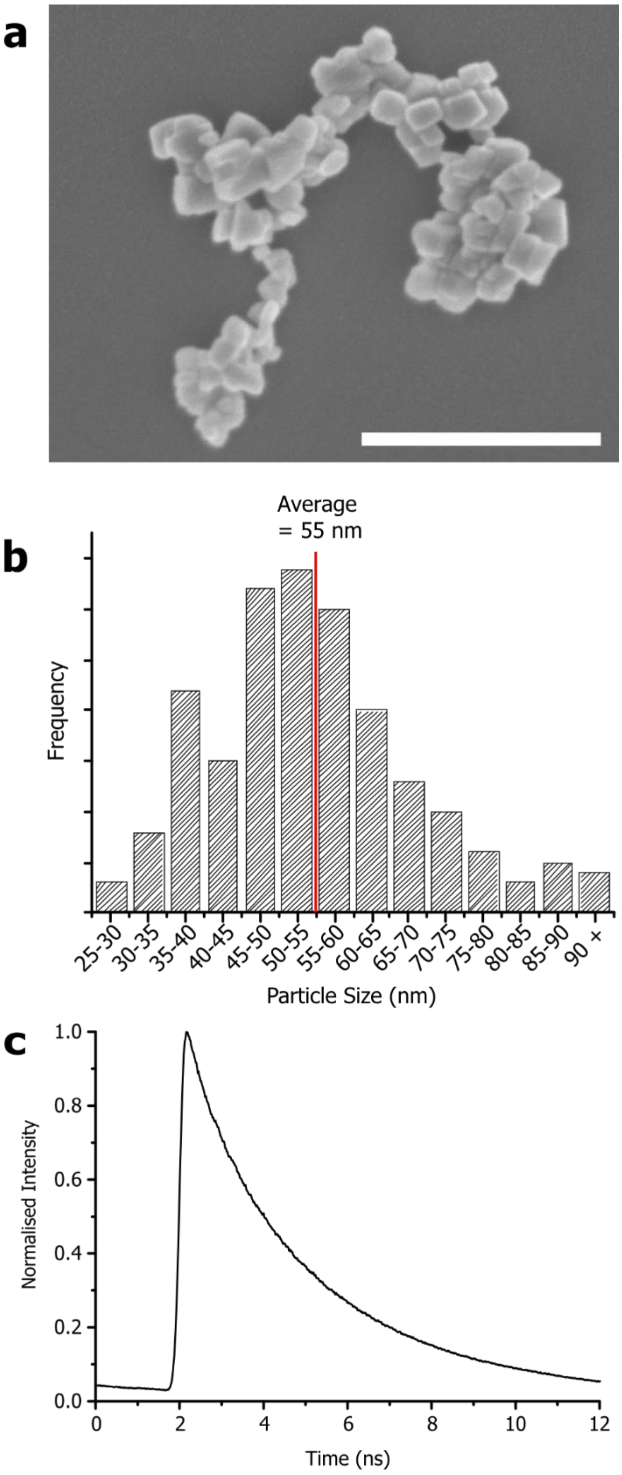

Supplementary Figure 16: Representative SEM micrograph of calcite nanoparticles (**a**) and size distribution histogram of calcite nanoparticles as imaged by SEM (sample size = 300) (**b**). The average value, 55 nm, has been marked in red. Average fluorescence lifetime plot for GREEN/nanocalcite composites, where τ = 3.0 ns (**c**). Scale bar: a) 500 nm

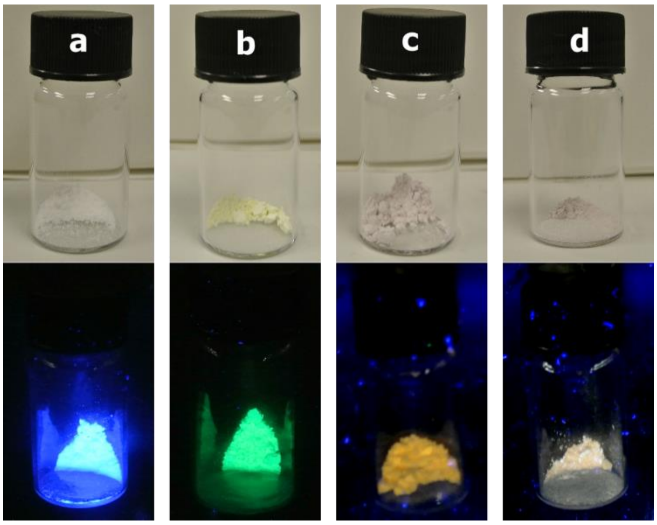

Supplementary Figure 17: Dry fluorescent dye/calcite nanoparticles occluding **a**) BLUE, **b**) GREEN, **c**) RED and **d**) dye mixture under normal light and UV light (365 nm).

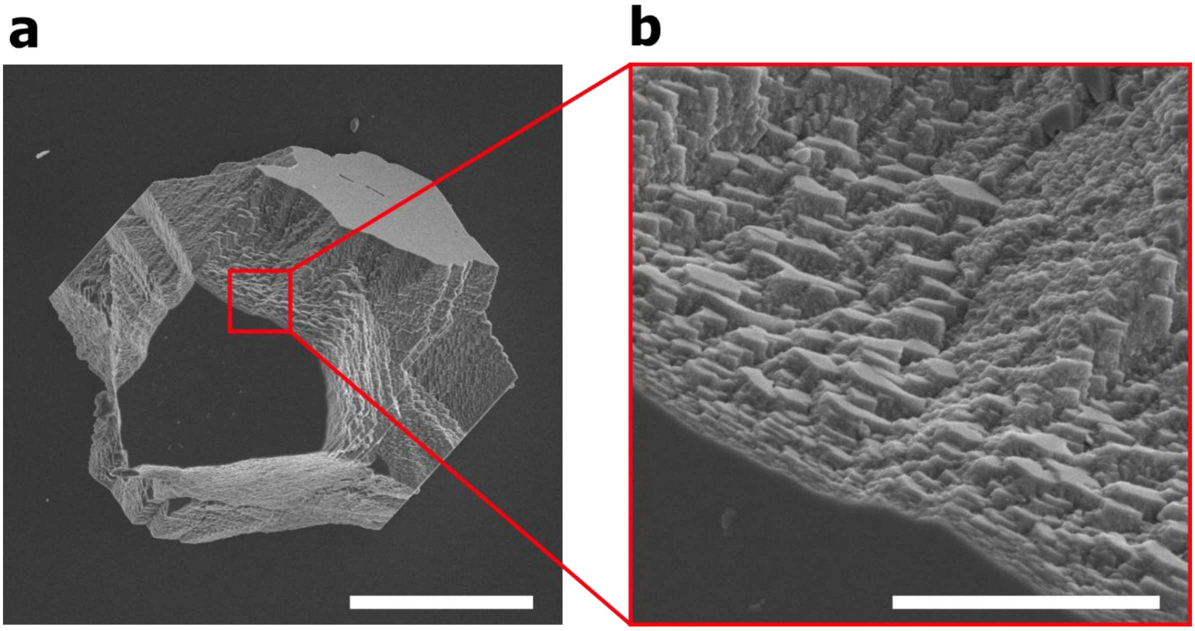

Supplementary Figure 18: **a**) A calcite crystal grown under conditions of [Ca^{2+}] = [HCO_3^-] = 3.5 mM with [GREEN] = 0.02 mM (as seen in **Figure 3**) and **b**) a higher magnification micrograph of the image boxed in red in **a** demonstrating the highly roughened surfaces formed. Higher magnification micrographs reveal that new “faces” formed due to the presence of an additive are simply disrupted {104} faces. Scale bar: main) 20 µm, inset)

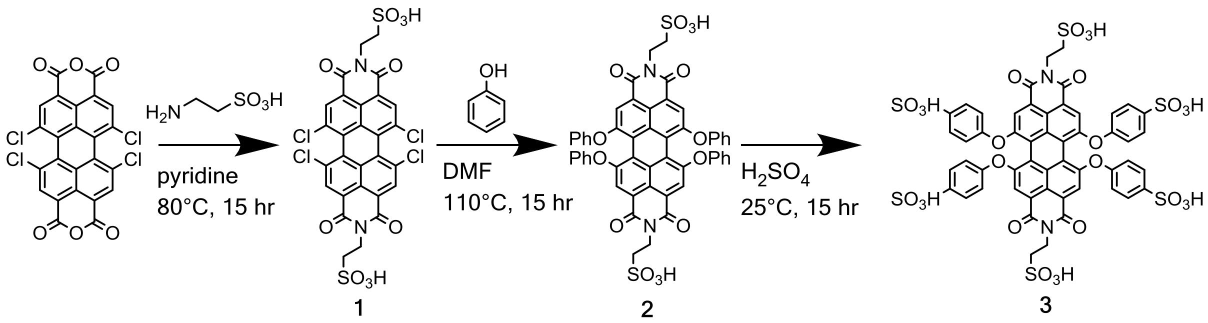

Supplementary Figure 19: Reaction scheme for the synthesis of RED, a red-emitting fluorescent dye. Specific details are provided in the Methods section.

Supplementary Tables

	Mixture				
	**1**	**2**	**3**	**4**	**5**
**BLUE (mM)**	0.005	0.005	0.005	0.01	0.01
**GREEN (mM)**	0.1	0.1	0.2	0.2	0.2
**RED (mM)**	0.002	0.005	0.005	0.002	0.005
**Ratio (BLUE:GREEN:RED)**	5:100:2	1:20:1	1:40:1	5:100:1	2:40:1

Supplementary Table 1: Candidate BLUE:GREEN:RED mixtures examined in calcite growth studies, given by concentration and the molar ratio.

Dye	$[Ca^{2+}]$ (mM)	$[Dye]/[Ca^{2+}]$	mol% dye	wt% dye
**BLUE**	157	0.000064	0.0178	0.0617
**GREEN**	157	0.00064	0.0090	0.0472
**RED**	157	0.0000064	0.0013	0.0164

Supplementary Table 2:Quantification of fluorescent dye incorporated in calcite nanoparticles.
